# Entomological Survey and *Leishmania* (*Leishmania*) *mexicana* Prevalence in Sand Fly Species during an Outbreak of Cutaneous Leishmaniasis in Quintana Roo State, Mexico

**DOI:** 10.3390/tropicalmed8100465

**Published:** 2023-10-05

**Authors:** Isabel C. Cañeda-Guzmán, Ana C. Montes de Oca-Aguilar, Carlos I. Miranda-Caballero, Estefania Grostieta, Fabián Correa-Morales, Raquel Romero-Pérez, Francisco E. Romero-Contreras, José A. Rodríguez-Atanacio, Karina Ruiz-Tovar, Herón Huerta, Pedro. C Mis-Avila, Marco R. Quintanilla-Cedillo, Miguel A. Lammoglia-Villagómez, Selene Blum-Domínguez, Paulino Tamay-Segovia, Rebeca Rojas-Ronquillo, Sokani Sánchez-Montes, Ingeborg Becker

**Affiliations:** 1 Centro de Medicina Tropical, División de Investigación, Facultad de Medicina, Universidad Nacional Autónoma de México, Mexico City 06720, Mexico; icanedag@ciencias.unam.mx (I.C.C.-G.); miranda-caballero@ciencias.unam.mx (C.I.M.-C.); estefania.grostieta@ciencias.unam.mx (E.G.); 2 Laboratorio de Inmunología, Centro de Investigaciones Regionales “Dr. Hideyo Noguchi”, Universidad Autónoma de Yucatán, Mérida, Yucatán 97000, Yucatán, Mexico; t-amontes.aguilar@correo.uady.mx; 3 Programa de Enfermedades Transmitidas Por Vectores, Centro Nacional de Programas Preventivos y Control de Enfermedades, Secretaría de Salud, Mexico City 11800, Mexico; fabian.correa@salud.gob.mx (F.C.-M.); raquel.romero@salud.gob.mx (R.R.-P.); francisco.romero@salud.gob.mx (F.E.R.-C.); insectopia@me.com (J.A.R.-A.); 4 Laboratorio de Entomología, Instituto de Diagnóstico y Referencia Epidemiológicos ‘Dr, Manuel Martínez Báez’, Secretaría de Salud, Mexico City 01480, Mexico; karina.ruiz@salud.gob.mx (K.R.-T.); heron.huerta@salud.gob.mx (H.H.); 5 Departamento de Enfermedades Transmitidas por Vector y Zoonosis, Servicios Estatales de Salud de Quintana Roo, Chetumal Quintana Roo 77000, Mexico; 6 Clínica Carranza, Chetumal Quintana Roo 77035, Mexico; dermachetumal@prodigy.net.mx; 7 Facultad de Ciencias Biológicas y Agropecuarias, Región Tuxpan, Universidad Veracruzana, Tuxpan de Rodríguez Cano, Veracruz 92870, Mexico; mlammoglia@uv.mx (M.A.L.-V.); rebrojas@uv.mx (R.R.-R.); 8 Laboratorio de Enfermedades Tropicales, Centro de Investigaciones Biomédicas de la Universidad Autónoma de Campeche, Campeche 24039, Mexico; selcblum@uacam.mx; 9 Laboratorio de Enfermedades Transmitidas por Vectores y Zoonosis, Centro de Investigaciones Biomédicas de la Universidad Autónoma de Campeche, Campeche 24039, Mexico; pautamay@uacam.mx

**Keywords:** parasite, neglected tropical disease, sand fly, Yucatan Peninsula

## Abstract

**(1) Background**: Localized cutaneous leishmaniasis is a neglected vector-borne disease that has become a serious public health problem in the Yucatan Peninsula. Although more than 60% of cases originate from the state of Quintana Roo, it is one of the least explored areas in terms of incriminating vectors of the Leishmania parasite. Additionally, cases of leishmaniasis have increased substantially in that region in recent years. For this reason, we explored and provided primary evidence of Leishmania DNA in sand fly species from four localities during outbreaks of leishmaniasis in Quintana Roo. We also contributed information on the regional genetic diversity of Leishmania parasites. **(2) Methods**: Sand flies were collected during several periods from November 2022 to April 2023 using Mosquito Light Circle and Shannon traps, as well as an active entomological search in refuges. For *Leishmania* detection, we amplified a fragment of 300–350 bp of the internal transcribed spacer subunit 1 (ITS-1). **(3) Results:** Of the 242 females collected, we detected *Leishmania* DNA in 25 specimens represented by *Bichromomyia olmeca* (1), *Psathyromyia shannoni* (17), *Lutzomyia cruciata* (4), *Psathyromyia undulata* (2), and *Dampfomyia deleoni* (1). The detection of *Leishmania* in these last two species represents new records for the Yucatan Peninsula and for Mexico. *Leishmania* (*Leishmania*) *mexicana* was the only species detected in the Phlebotominae species, with prevalence values that ranked between 7.41% and 33.33% from specimens collected in the sylvatic areas of Cozumel Island and Petcacab. **(4) Conclusions:** This study provides the first evidence of infection of *Da*. *deleoni* and *Pa*. *undulata* by *L.* (*L*.) *Mexicana*. In addition, the presence of three dominant haplotypes in all the evaluated localities was evidenced using the analysis of genetic diversity, and the locality of Petcacab was the one with the circulation of two new haplotypes not previously described in Mexico or neighboring countries. These results highlight the importance of intensive epidemiological surveillance due to the dynamics of transmission of *Leishmania* between different species.

## 1. Introduction

Leishmaniasis is a neglected tropical disease (NTD) that causes significant morbidity and mortality in endemic areas, with a psychosocial impact on affected individuals and disability-adjusted life years lost in populations [1]. *Leishmania* parasites are the causal agents of these NTDs and have evolved to alternate between hosts (i.e., vertebrates) and susceptible vectors of the Phlebotominae subfamily [2]. Thus, the expansion of the geographic distribution and incidence of leishmaniasis in the world depends on both components (i.e., vectors and reservoirs). 

American cutaneous leishmaniasis is a serious public health problem in the neotropical region, since it is estimated that about 60, 941 cases occur each year, but the potential annual incidence is between 187,000 and 307,800 cases [3,4]. At least 20 species of *Leishmania* parasites are etiological agents of leishmaniasis across America [5]. Although the main risk of contracting leishmaniasis is associated with natural landscapes (i.e., sylvatic areas), evidence shows notable expansion and outbreaks of the disease in several neotropical countries, as well as drastic changes in transmission patterns that have given rise to emerging cycles in the urban and rural contexts [4]. Since vectors and reservoirs are sources of the circulating parasite, knowledge about the infection, behavior, and ecology of both components is essential to the establishment of any predictive expansion analysis and prevention strategy that minimizes human exposure to leishmaniasis disease. 

In Mexico, localized cutaneous leishmaniasis (LCL) has been recorded in 78% of the territory [6], and it is estimated that around 7.6 million people are at risk of transmission [3]. During the period 2010–2022 alone, a total of 8309 human cases were confirmed in the country [7]. Currently, five regions are recognized as endemic transmission areas, but the highest incidence and prevalence originate from the Yucatan Peninsula (YP) (>60% of cases [6]. In YP, the main parasite associated with LCL is *Leishmania* (*Leishmania*) *mexicana*, which maintains enzootic cycles in forest areas of the states of Campeche, Yucatan, and Quintana Roo. The main vector described for *Leishmania* transmission in YP is *Bichromomyia olmeca*. Nevertheless, four other species have been proposed as possible vectors in the region, making the identification of the parasite in different circulating species of vectors mandatory in order to understand the pattern of transmission [8,9]. 

In addition, the spatial distribution of the annual prevalence of LCL is not homogeneous in YP, since approximately 69% of cases originate from the Quintana Roo state. According to the national program for the control of vector-borne diseases, Quintana Roo state is experiencing a substantial increase in cases of LCL, from 16 cases in 2020 to 696 in 2022 [10]. Human cases have been reported in 10 of the 11 municipalities that comprise the state, with the municipalities of Felipe Carrillo Puerto (27.22%), Othon P. Blanco (19.64%), and Jose María Morelos (14.32%) accumulating the highest number of cases (Supplementary information Table S1). 

Nonetheless, in Quintana Roo state, the entomological research on LCL is limited to two works with different temporal scales [11,12]. In fact, there are more studies on *Leishmania* hosts [13,14,15,16] than on vector incrimination. In terms of vector fauna, the sylvatic areas of Quintana Roo harbor a high sand fly diversity, which represents more than 75% of the species registered in YP [16,17,18,19]. However, *Leishmania* infection has been documented only in three sand fly species, *Bi*. *olmeca* (Vargas & Díaz-Nájera), *Lutzomyia (Lu.) cruciata* Coquillett, and *Psathyromyia shanoni* Dyar [11,12]. On the other hand, although the parasite *L*. (*L*.) *mexicana* is the most prevalent species in the region, some studies document the presence of four other *Leishmania* species circulating among domestic animals [14,20], but so far, they have not been detected among the vector fauna and wild reservoirs. In addition, Quintana Roo is geographically close to Central American countries where the diversity of *Leishmania* parasites is greater [21]. 

For this reason, the aim of the present study was to explore and provide primary evidence of *Leishmania* prevalence in sand fly species from four localities with records of outbreaks of LCL in Quintana Roo, Mexico. Additionally, we contributed information on the regional genetic diversity of *Leishmania* parasites.

## 2. Materials and Methods

**Figure 1:**
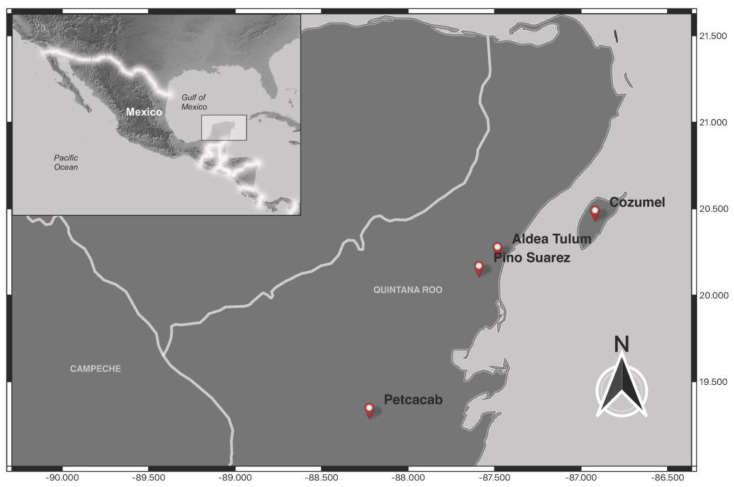
Sampling sites in the state of Quintana Roo, Mexico.

Due to an increase in LCL in the state of Quintana Roo, the National Program for the Control of Vector-Borne Diseases in accordance with national regulations (based on the Official Mexican Normativity NOM-032-SSA2-2014, for epidemiological surveillance, promotion, prevention, and control of diseases transmitted by vectors) of the National Center for Preventive Programs and Disease Control conducted punctual and directed fieldwork from November 2022 to April 2023 in four localities of Quintana Roo (Figure 1). 

Sand flies were collected during two consecutive nights with two Shannon traps (1.6 × 2.5 × 1.6 m) and two Mosquito Light traps (model 512; John W. Hock Co., Gainesville, FL, USA). Each night, the Shannon traps were randomly placed simultaneously in the forest closest to human settlements. Shannon traps were separated from each other by at least 100 m. Shannon traps were placed at 30 cm above the ground, and each one was baited by two persons protected by specialized clothing that prevents contact between people and sand fly species [22]. We employed glass aspirators to collect sand flies in Shannon Traps. On the other hand, automatic Mosquito Light traps were placed approximately 1 m above the ground. Both types of traps (Shannon and Light traps) were active for a 4 h period (18:00–21:00 h) per night. Furthermore, direct searches of different ecotopes associated with daytime resting sites of sand flies (i.e., trunks, natural holes, etc.) were conducted. All specimens collected were stored in 70% ethanol. Each female was separated into head, thorax, and posterior segments of the abdomen (containing the spermatheca). Only head and posterior abdominal segments were mounted on permanent slides using protocol by Ibañez-Bernal [23]. Other specimens were mounted semi-permanently using glycerin, distilled water, and Arabic gum (1:2:2) [24]. Sand flies were identified according to morphological characteristics (genitalia, antenna, and cibarium) using the specialized taxonomic keys of Young and Duncan [25], Ibáñez-Bernal [23,26].

Genomic DNA was extracted individually from the thorax and remaining segments of the abdomen of all specimens using 500 µL of a 10% solution of Chelex-100 and 20 µL of proteinase K [27]. The samples were incubated at 56 °C for 12 h, and the supernatant containing the genetic material was recovered in new tubes. For *Leishmania* species identification, the sample was tested for the amplification of a ~300–350 bp fragment of the internal transcribed subunit 1 (ITS1) [28]. The thermal conditions were as follows: an initial cycle of denaturation at 94 °C for 10 min, 35 cycles at 94 °C for 30 s, 53 °C for 30 s, and 72 °C for 45 s, with a final extension of 72 °C for 5 min.

The reaction mixture was prepared in a final volume of 25 µL with 12.5 µL of GoTaq^®^ Green Master Mix, 2X, Promega Corporation (Madison, WI, USA), 1 µL of each primer (100 ng each), 10 µL DNA (~50 ng), and 0.5 µL nuclease-free water. We included DNA of *L. (L.) mexicana* strain Lacandona as a positive control, and nuclease-free ultrapure water instead of DNA as a negative control. The amplification products were subjected to electrophoresis in 2% agarose gels, stained with Smartglow. All PCR-positive products were sequenced at Macrogen, Korea.

The obtained sequences were subjected to local alignments using the Nucleotide Basic Local Alignment Search Tool [BLASTn]. To corroborate the identity of the parasite recovered, we obtained sequences of validated species of the genus Leishmania deposited in GenBank and performed a phylogenetic reconstruction. For this, a global alignment was performed using ClustalW present in MEGA 6. The best substitution model was selected using the lowest BIC (Bayesian Information Criterion) value. Subsequently, a phylogenetic inference analysis was performed using the maximum likelihood algorithm, also with MEGA 6. The support of the branches was evaluated using 10,000 Bootstrap replicates. Gaps were excluded from the analysis.

Additionally, in order to ascertain genetic diversity, we estimated the number of haplotypes, number of unique haplotypes, number of mutations, number of segregating sites, number of unique sites, haplotypic diversity, and nucleotide diversity in DNAsp 5.10 [29]. To identify the relationship among haplotypes, minimal union networks were constructed using the program PopArt.

## 3. Results

A total of 242 females belonging to six genera and seven species (Bi. olmeca, Dampfomyia (Coromyia) deloni (Fairchild and Hertig), Lutzomyia (Lutzomyia) cruciata Coquillet, Pintomyia (Pifanomyia) serrana, Psathyromyia (Psathyromyia) shannoni (Damascenco and Arouck), Psathyromyia (Psathyromyia) undulata (Fairchild and Hertig), and Psychodopygus panamensis (Shannon)) were recorded from four localities of Quintana Roo state (Table 1). Most of the sand fly species were collected in Shannon traps (45.87%), followed by Mosquito Circle traps (44.63%). Only 23 sand flies were collected with direct searches (AES, Table 1). 

The species with the highest number of specimens collected was *Pa*. *shannoni* with 157 specimens, followed by *Lu*. *cruciata* with 27. Singletons of *Pi*. *serrana* and *Ps*. *panamensis* were recorded. Petcacab was the locality with the highest species richness (n = 7), followed by Aldea Tulum (n = 4). The species with the widest distribution in three of the four collection sites was *Pa*. *undulata*. Only 17 engorged specimens were collected, and *Pa*. *undulata* and *Pa*. *shannoni* presented the highest number of specimens, with eight of each one. 

**Figure 2:**
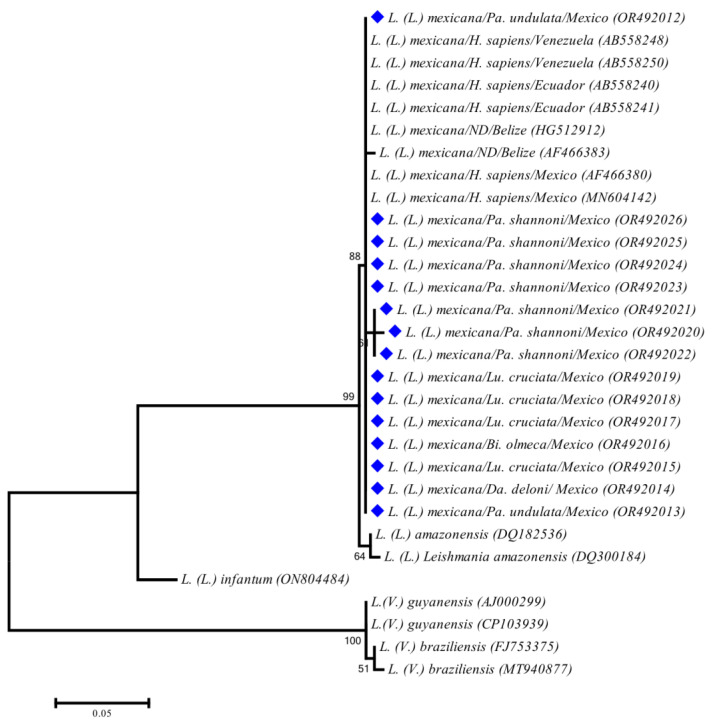
Maximum likelihood (ML) phylogenetic tree of *Leishmania* parasites generated using a fragment of 310 bp of the internal transcribed spacer 1 (ITS1) based on the General Time Reversible model with a discrete gamma distribution (+G). Sequences generated in this study are marked with a blue diamond in the figure.

*Leishmania* DNA was detected in 25 specimens of five species from two localities, Cozumel and Petcacab. In general, the prevalence ranged from 7.41% to 33.33% (Table 1), where *Pa*. *shannoni* and *Lu*. *cruciata* were the sand fly species with the highest numbers of specimens positive for *Leishmania*. Although to a lesser extent, we recorded for the first-time infection positivity for *Leishmania* in *Pa*. *undulata* and *Da*. *deleoni*. Only one specimen of the proven vector *Bi*. *olmeca* was positive for the parasite. We recovered 15 sequences of 310 bp of *Leishmania* ITS-1. According to the similarity analysis, all the recovered sequences exhibited 99%–100% (305/307 and 307/307 bp) of matches with *L*. (*L*.) *mexicana* from Mexico and Ecuador (GenBank accession numbers MN604142.1 and AB558241.1, respectively). Similarly, the phylogenetic inference based on maximum likelihood clustered our sequences within the monophyletic clade of *L*. (*L*.) *mexicana* with a high support value (88%) (Figure 2).

**Figure 3:**
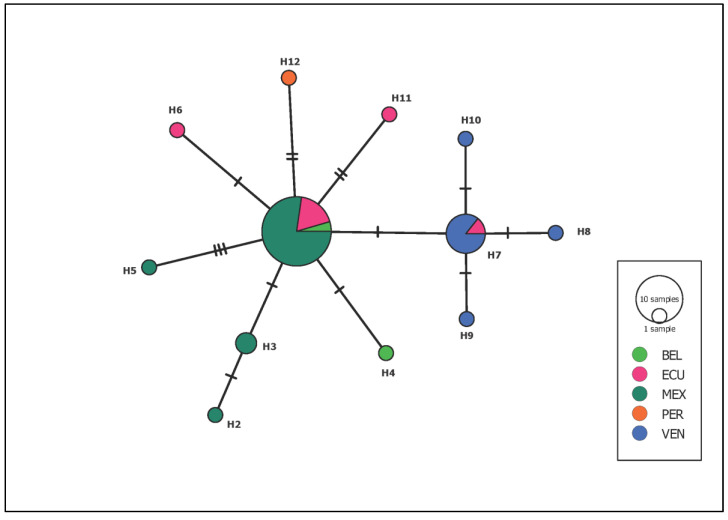
Minimal union network of *L*. (*L*.) *mexicana* from five different populations in Latin America, based on sequences of 300 bp of the ITS-1. BEL: Belize; ECU: Ecuador; MEX: Mexico; PER: Peru; and VEN, Venezuela.

For genetic diversity analysis of *Leishmania*, we aligned 15 sequences recorded in this study and 25 sequences previously deposited in GenBank (2 from Belize, 7 from Ecuador, 6 from México, 1 from Peru, and 9 from Venezuela). The final alignment consisted of 310 bp, with 288 conserved and 23 variable sites, 16 singletons and 6 parsimony-informative sites. We detected the presence of 12 haplotypes (Figure 3). 

The most frequent haplotype detected was H1 with 22 sequences (55%), followed by haplotype H7 with 7 (17.5%) and H3 with 2 (5%). Haplotype diversity (Hd) was 0.6756, and nucleotide diversity was 0.00507. Particularly, in the case of the samples from Quintana Roo, we detected the presence of three haplotypes, and the locality of Petcacab being the only one that registered the presence of all of them. Additionally, this locality accounted for the presence of two unique haplotypes not previously reported in Mexico, Central America, or South America, as per our knowledge.

## 4. Discussion

This research provides information on the potential participation of five sand fly species in LCL transmission cycles in one of the least explored areas in YP. All the species recorded in the present study had already been documented previously in Quintana Roo [17,19]. However, it is important to highlight that until our discovery, the local fauna of sand fly species and the circulation of the parasite *L*. (*L*.) *mexicana* on Cozumel Island was completely unknown. Although with low abundances, the detection of two specimens of *Pa*. *undulata* infected by *Leishmania* opens new research opportunities regarding the historical processes that have generated the diversity of local sand fly species, as well as characterizes the different eco-epidemiological components that would be participating in the transmission of the *Leishmania* parasite on this Caribbean Island.

We did not detect the presence of other *Leishmania* species in the sand fly fauna. Furthermore, all sand fly species were positive for *L*. (*L*.) *mexicana* DNA, which supports all studies on infection in vectors and wild reservoirs in the region [8,9,12,30,31]. Additionally, this work provides the first sequences of *L*. (*L*.) *mexicana* for the state of Quintana Roo, which are essential for the analysis of genetic diversity at the border of Mexico and in two Central American countries. Our findings provide a preliminary overview of the intra-specific variability of *L*. (*L*.) *mexicana* based on haplotype analysis. Interestingly, the analysis of genetic diversity of the parasite demonstrated the presence of a dominant haplotype present in all localities and all infected sand fly species, which could suggest the presence of possible vertebrate reservoirs that maintain this haplotype in the environment so that multiple species of sand flies become infected [8,9,30,31]. Also, we identified that the town of Petcacab presented the greatest haplotype diversity, with three and two of them being unique and previously undetected in Mexico or neighboring countries. This may be due to the migration of the parasite vectors from local sylvatic cycles. Although speculative, this could be associated with diverse species compositions of sand flies from different feeding guilds (strict zoophiles, i.e., *Da*. *deleoni*, and generalists, i.e., *Lu*. *cruciata*), which allow them to be in contact with larger numbers of parasite haplotypes that circulate in other mammalian species that make up the vertebrate assemblage in the region. This is of vital importance for monitoring the movement of the parasite among them and establishing the effect that control measures could have on the reduction of genetic variants, as well as possible recombination events, that have been reported in other Central American countries.

In general, our range of infection by *L*. (*L*.) *mexicana* detected in Phlebotomine sand flies from Quintana Roo state (7.41%–33.33%) is similar to the historical prevalence documented in the five species (i.e., *Psy. panamensis* (Shannon), *Nyssomyia ylephyletor* (Fairchild and Hertig), *Bi*. *olmeca*, *Lu*. *cruciate,* and *Pa*. *shannoni*) of medical importance recognized so far in YP (0.2%–43.8%) (8,9,30,31). Furthermore, our findings on the detection of *L*. (*L*.) *mexicana* in the highly anthropophilic sand flies *Bi*. *olmeca*, *Pa*. *shannoni,* and *Lu*. (*Lu*.) *cruciata* reinforce the notion that all these species are important vectors in YP [8,9] and probably in other regions of Mexico where LCL is also prevalent. The latter is supported by studies showing that the geographic occurrence of both *Pa. shannoni* and *Lu*. (*Lu*.) *cruciata* better explains the leishmaniasis distribution pattern in the country, than that of the proven vector *Bi*. *olmeca* [32,33]. Although faunistic studies performed in Quintana Roo have showed a strong dominance of *Pa*. *shannoni* and *Lu*. (*Lu*.) *cruciata* in several foci of LCL [17,18,34]. Previously, the only evidence of records of *L*. (*L*). *mexicana* infection in both sand fly species were obtained from specimens collected in the peri-urban area of Chetumal city [12]. Notably, our results show a prevalence between 8.78% and 31.38%, higher than those reported previously in the region [12]. This is even more evident for *Bi*. *olmeca*, which reports a prevalence of 1.5% in the peri-urban area, whereas in the forest surrounding Petcacab, the values were higher at 31.83%. In addition to the sampling effort, several non-mutually exclusive factors can explain these disparities in prevalence. For example, the landscape context in our study is completely different to that of the peri-urban area, and this may influence the structure and abundance of the vertebrate community, which in turn modulates the prevalence patterns of the *Leishmania* parasite [35]. However, in the YP and specifically in Quintana Roo, there are still some aspects related to the study of the vector–*Leishmania*–host interaction that need to be addressed.

Our current research provides the first evidence of infection of *Da*. *deleoni* and *Pa*. *undulata* by *L* (*L*). *mexicana.* Although both species have occasionally been caught on human bait [24,36,37,38], some studies outline them as sand flies with a marked rodentophilic behavior [37,39,40]. The latter is important because the three main reservoirs of *L*. (*L*.) *mexicana* (*Peromyscus yucatanicus*, *Ototylomys phyllotis,* and *Heteromys gaumeri*) are rodents widely distributed in the YP [41,42]. There is strong evidence that *Da*. *deleoni* is the dominant zoophilic species in the region [17,18,43,44,45], and yet, the presence of parasite DNA had never been explored. In this study, we found only one specimen that tested positive for *Leishmania,* but our findings contrast those of William [36] who, in a study focused on LCL in Belize, dissected more than 1622 females but never found infected specimens. In relation to *Pa*. *undulata*, in general, the natural history of this sand fly species remains unknown. However, in Guatemala, there are records of females infected with flagellated unidentified parasites [24]. In our country, while faunistic studies record low abundances of *Pa*. *undulata* [17,38,46,47,48], ecological niche models predict a wide geographic range [33] that coincides with ecoregions with active transmission of leishmaniasis [32]. Nevertheless, our results are not conclusive regarding whether both species (*Pa*. *undulata* and *Da*. *deleoni*) participate in the enzootic cycles of *L* (*L*.) *mexicana*, but reinforce the importance of conducting studies that evaluate their spatial and temporal interactions with the *Leishmania* species and wildlife in the region and other Mesoamerican areas. 

Assuming that LCL cases are increasing in several regions of the YP [49,50] and particularly in Quintana Roo state, entomological surveys and vector incrimination studies need to be intensified. Although our study has some limitations, including a period of non-systematized collections, our data support the idea that *L*. (*L*.) *mexicana* is the most prevalent parasite and that *Lu*. (*Lu*.) *cruciata* and *Pa*. *shannoni* together with *Bi*. *olmeca* are the most important vectors of this species. Similarly, we contribute to the existing knowledge of the potential enzootic cycles of the parasite by detecting *Da*. *deleoni* and *Pa*. *undulata* infected with *L* (*L*.) *mexicana* in the region. We hope that our findings motivate the development of lines of research that allow the identification of the different components of the natural cycle of the parasite, as well as the ecological and social factors associated with changes in the prevalence and incidence of LCL in this and other states that comprise the YP.

## Figures and Tables

**Table 1:** Relationship of sand fly species collected and positive for *L*. (*L*.) *mexicana* in the state of Quintana Roo, Mexico. n: abundance; SH: Shannon trap; MLT: Mosquito Light Circle; AES: active entomological surveillance; +: positive samples; and %: prevalence.

Locality	Latitude	Longitude	Date	Species	n	SH	MLT	AES	Feeding	+	%
Aldea Tulum	20°13′11.246″ N	87°29′2.018″ O	12–13 April 2023	*Bichromomyia olmeca*	3	3	0	0	0	0	0
				*Lutzomyia cruciata*	5	3	2	0	0	0	0
				*Psathyromyia shannoni*	5	3	2	0	0	0	0
				*Psathyromyia undulata*	1	1	0	0	0	0	0
Cozumel	20°25′54.331″ N	86°55′12.988″ O	20 November 2023	*Psathyromyia undulata*	23	0	0	23	7	2	7.41
Petcacab	19°17′24.215″ N	19°17′24.215″ O	21–22 March 2023	*Dampfomyia deloni*	7	1	6	0	0	1	14.29
				*Bichromomyia olmeca*	3	3	0	0	1	1	33.33
				*Lutzomyia cruciata*	22	11	11	0	0	4	18.18
				*Pintomyia serrana*	1	1	0	0	0	0	0
				*Psthyromyia shannoni*	152	84	68	0	8	17	11.18
				*Psathyromyia undulata*	1	0	1	0	1	0	0
				*Psychodopygus panamensis*	1	1	0	0	0	0	0
Pino Suárez	20°6′37.427″ N	87°35′28.346″ O	13 April 2023	*Lutzomyia cruciata*	18	0	18	0	0	0	0
				**Total**	242	111	108	23	17	25	10.33

## Data Availability

The data that support the results of this study are available in GenBank, under the following accessions numbers OR492012-OR492026.

## Supplementary Materials

The following supporting information can be downloaded at: https://www.mdpi.com/article/10.3390/tropicalmed8100465/s1, Table S1: Distribution of LCL cases in the municipalities of the state of Quintana Roo during the period 2020-2023. Data according to Health Services of the state of Quintana Roo.
