# Supplementary material for: Entomological Survey and Leishmania (Leishmania) mexicana Prevalence in Sand Fly Species during an Outbreak of Cutaneous Leishmaniasis in Quintana Roo State, Mexico
Source: Trop Med Infect Dis. 2023 Oct 5;8(10):465. doi: 10.3390/tropicalmed8100465 (PMC10610947; doi:10.3390/tropicalmed8100465)
Supplement: Supplementary file 1 [file tropicalmed-08-00465-s001.zip › tropicalmed-2613156-supplementary.pdf]

Table S1. Distribution of LCL cases in the municipalities of the state of Quintana Roo during the period 2020-2023. Data according to Health Services of the state of Quintana Roo.

**Municipality**	**Year**				**Total**	**%**
	**2020**	**2021**	**2022**	**April 2023**		
Cozumel	0	0	0	1	1	0.06
Felipe Carrillo Puerto	4	58	157	201	420	27.22
Isla Mujeres	0	0	4	0	4	0.26
Othon P. Blanco	6	72	147	78	303	19.64
Benito Juárez	1	18	90	112	221	14.32
José María Morelos	5	28	78	22	133	8.62
Lázaro Cárdenas	0	10	50	26	86	5.57
Solidaridad	0	15	47	81	143	9.27
Tulum	0	22	67	43	132	8.55
Bacalar	0	12	56	32	100	6.48
**Total**	16	235	696	596	1543	100.00
